# Reactive infectious mucocutaneous eruption – repeat etanercept after intravenous immunoglobulin: A case report

**DOI:** 10.1177/2050313X221117887

**Published:** 2022-08-17

**Authors:** Rochelle Tonkin, Malika Ladha, Nicole Johnson, William F Astle, Ami Britton, Neil H Shear, Luis Murguía-Favela, Michele Ramien

**Affiliations:** 1 Division of Dermatology, Department of Medicine, University of Calgary, Calgary, AB, Canada; 2 Department of Dermatology, University of Toronto, Toronto, ON, Canada; 3 Sunnybrook Health Sciences Centre, Toronto, ON, Canada; 4 Section of Rheumatology, Department of Pediatrics, Cumming School of Medicine, University of Calgary, Calgary, AB, Canada; 5 Pediatric Ophthalmology, Vision Clinic, Alberta Children’s Hospital, Department of Surgery, University of Calgary, Calgary, AB, Canada; 6 Wound Care/Surgery Clinic Nurse, Department of Pediatrics, Alberta Children’s Hospital, University of Calgary, Calgary, AB, Canada; 7 Section of Hematology and Immunology, Department of Pediatrics, Alberta Children’s Hospital, University of Calgary, Calgary, AB, Canada; 8 Division of Community Pediatrics (Dermatology), Department of Pediatrics, Alberta Children’s Hospital, University of Calgary, Calgary, AB, Canada

**Keywords:** Etanercept, intravenous immunoglobulin, reactive infectious mucocutaneous eruption, mycoplasma-induced rash and mucositis, Coronavirus disease 2019

## Abstract

Reactive infectious mucocutaneous eruption is a recently distinguished mucosal-predominant blistering eruption triggered by respiratory infections. We describe a previously healthy 11-year-old Black female with rapidly progressive mucocutaneous blistering after prodromal respiratory infection symptoms. Reactive infectious mucocutaneous eruption was suspected and treated with systemic corticosteroids followed by etanercept. Twenty-four hours after etanercept, the diagnosis of multisystem inflammatory syndrome in children was raised and intravenous immunoglobulin was given. Rapidly worsening mucocutaneous disease ensued but was controlled by a second dose of etanercept. Our case highlights the following: (1) the novel observation of possible interaction/neutralization of etanercept by intravenous immunoglobulin, (2) the challenging differential diagnosis of multisystem inflammatory syndrome in children for reactive infectious mucocutaneous eruption patients in the Coronavirus disease 2019 (COVID-19) pandemic, and (3) the role of early treatment to prevent dyspigmentation.

## Introduction

Reactive infectious mucocutaneous eruption (RIME) describes an eruption of prominent mucositis with/without cutaneous involvement triggered by *Mycoplasma pneumoniae* (MP) and other infectious causes.^1,2^ The treatment approach is twofold: eliminate the inciting infection and halt mucocutaneous progression. The latter can be achieved with immunomodulatory agents, including corticosteroids, intravenous immunoglobulin (IVIG), cyclosporine, or tumour necrosis factor (TNF)-α inhibitors.^1,3–5^ We report a case of RIME that responded rapidly to etanercept (ETN) but worsened with IVIG administration, requiring a second ETN dose.

## Case report

A previously healthy 11-year-old Black female presented to the emergency department with rapidly progressive mucocutaneous blisters and pharyngitis-like symptoms over 24 h. This was preceded by 4 days of fever, malaise, headache, conjunctivitis, odynophagia, abdominal pain, emesis, and dysuria. She had no prior medication or known infectious exposures.

**Figure 1.**
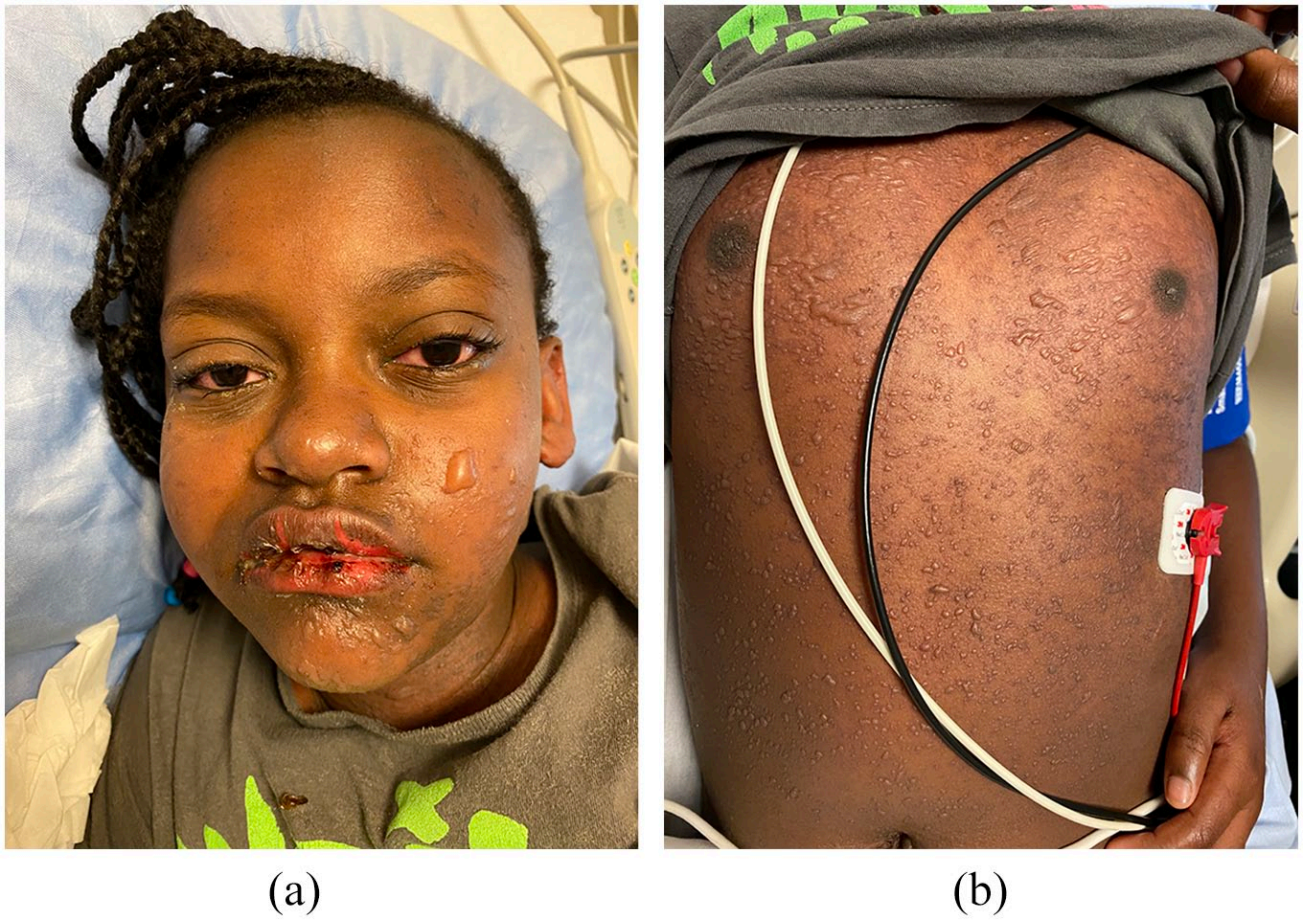
(a and b) Facial and truncal blistering, and hemorrhagic oral mucositis at initial presentation (20% BSA involvement).

On examination, she was febrile and tachycardic with diffuse abdominal tenderness. Multiple well-defined tense clear fluid-filled vesicles/bullae (1–10 mm in size) presented on the face, trunk, upper/lower proximal extremities, and labia majora (Figure 1(a) and (b)), affecting 20% of the body surface area (BSA). The vermillion lip had skin sloughing with hemorrhagic crusting, and the oral mucosa was diffusely eroded. The genital mucosa had marked erythema and erosions without extension to the anal mucosa. Nikolsky and Asboe-Hansen signs were negative. Despite non-purulent ocular exudate with scleral conjunctival erythema, there was no evidence of ocular complications.

Blood work demonstrated lymphocytopenia along with elevated transaminases and inflammatory markers. Coronavirus disease 2019 (COVID-19) (including serology) and respiratory infection assays were negative. MP immunoglobulin-M was indeterminate. Chest X-ray, cardiac investigations, and tests for autoimmune connective tissue disease were normal. Skin biopsies demonstrated vacuolar interface dermatitis and superficial dermal perivascular inflammatory infiltrate consistent with erythema multiforme (EM)/Steven–Johnson syndrome (SJS)/toxic epidermal necrolysis (TEN). Perilesional direct immunofluorescence was negative.

She was diagnosed with RIME with an unidentified viral trigger (based on prodromal symptoms). Differential diagnoses included drug-induced SJS (no drug exposures within the preceding 9 months), multisystem inflammatory syndrome in children (MIS-C; negative COVID-19; no contacts), toxic shock syndrome (unlikely with extensive mucosal involvement), and Kawasaki disease (criteria not met).

**Figure 2.**
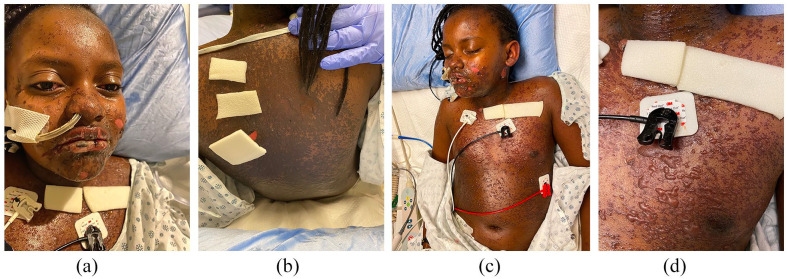
(a–d) Progression of tense cutaneous blisters and hemorrhagic oral mucositis 24 h post-IVIG on day 4 (35% BSA involvement), prior to which she had received methylprednisolone and one dose of ETN.

**Figure 3.**
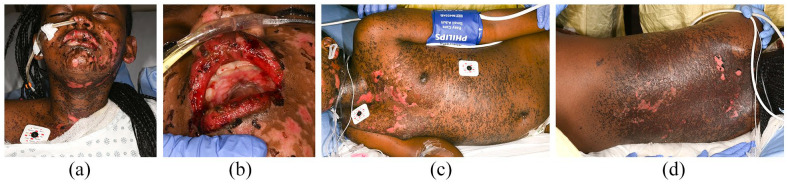
Resolution of tense blisters, drying of erosions, and halted progression after receiving the second dose of ETN subsequent to IVIG administration given 48 h prior. (a) Facial blistering with erosions and hemorrhagic oral mucositis 24 h after the second dose of ETN on day 6. (b–d) Hemorrhagic oral mucositis and diffuse vesicles and bullae on the trunk with skin sloughing 48 h after the second dose of ETN on day 7.

A single dose of methylprednisolone (30 mg/kg/dose intravenous (IV)) was given on day 1, followed by a dose of ETN (0.6 mg/kg subcutaneous (SC)) 24 h later. Subsequently, her skin and mucosal lesions stabilized. During handover, rheumatology suggested the diagnosis of MIS-C (COVID-19 serology was pending); therefore, one dose of IVIG (2 g/kg IV) was administered 24 h after ETN (day 3). Her mucocutaneous lesions rapidly worsened within 24 h (to 35% BSA involvement post-IVIG (Figure 2(a)–(d)) from 20% pre-IVIG) prompting a second higher dose of ETN (0.8 mg/kg SC) on day 5. The following day, she demonstrated improvement with drying of erosions and halted progression (Figure 3(a)–(d)).

Her hemorrhagic oral mucositis was treated with a compounded mouthwash known as Akabutu’s (Table 1),^6^ sucralfate suspension and tranexamic acid gel. Clobetasol propionate 0.05% ointment and lidocaine 2% gel were applied to oral/genital lesions. Petrolatum and non-adhesive dressings were applied to eroded skin. Supportive management included empiric antibiotic therapy, multimodal analgesia, lubricating/dexamethasone ophthalmic drops, urinary catheter, nasogastric tube, and a proton pump inhibitor.

**Table 1.** Akabutu’s compounded mouthwash (developed by Dr John Akabutu, Pediatric Oncologist in Edmonton, Alberta, for mucositis symptom relief.^6^)

Ingredients
Nystatin 100,000 units/mL × 42 mL Lidocaine HCl viscous 2% × 50 mL Sodium chloride 0.9% × 200 mL Hydrocortisone 10 mg × 5 tabs Glycerin 100% × 4 mL
Directions for use
Swish/gargle 15–30 mL in mouth/throat for 1 min, and then spit out excess. Repeat every 4–6 h as needed. Avoid eating or drinking for approximately 30 min after use.

**Figure 4.**
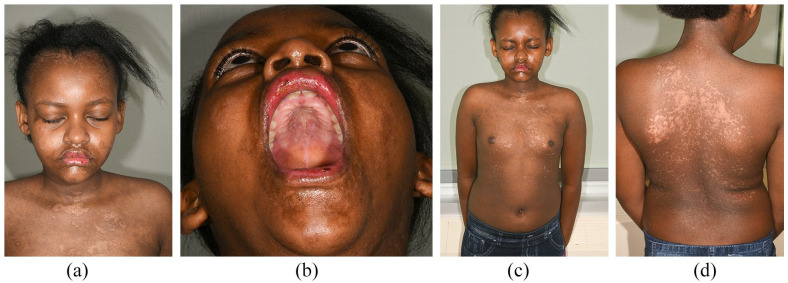
Follow-up post-discharge 3 weeks after receiving second dose of ETN. (a and b) There is almost complete resolution of mucocutaneous lesions with only mild desquamation of vermillion lip remains. (c and d) Re-epithelialization has occurred in areas of previous blisters/erosions with minimal dyspigmentation.

Two weeks after the second ETN dose and supportive care, she was discharged home (day 20). Areas of previous blisters had re-epithelialized with post-inflammatory hypo-/hyper-pigmentation, and her mucositis had greatly improved. One-week post-discharge, she had almost complete recovery with minimal dyspigmentation and only mild vermillion lip desquamation remaining (Figure 4(a)–(d)).

## Discussion

RIME unifies mycoplasma-induced rash and mucositis (MIRM) and other clinically similar mucosal-predominant eruptions related to a growing number of viral infections, including COVID-19.^2,7–12^ RIME presents with a prodrome of respiratory symptoms during its 2- to 3-week incubation period. Nearly one-quarter of MP pneumonia patients develop mucocutaneous disease, while RIME manifests in 7%.^13^

The morphology (Table 2), pathophysiology, and outcomes of RIME are distinct from SJS/TEN (rapidly progressive skin necrosis with mucositis; most often drug-induced) or EM (typical target papules).^1^ Mucous membranes are more severely affected than skin in RIME. Skin lesions are often sparse (47%) or absent (34%), and rarely extensive.^1^ Although investigations did not confirm the precise infectious pathogen, our patient met criteria for RIME (Table 3) based on her prodromal symptoms and clinical morphology. Acute-onset mucosal-predominant eruption with prodromal respiratory illness and without relevant drug exposure in children is most likely RIME.^14^

**Table 2.** Mucocutaneous morphology of RIME.

Lesion type	Clinical findings
Mucosal^1,15^	• Oral: erosions, ulcers, vesiculobullae, denudation, and hemorrhagic crusting • Ocular: conjunctival injection, conjunctivitis, photophobia, eyelid oedema, lid margin ulceration, conjunctival pseudomembranes, and rarely corneal erosions/ulcers • Urogenital: erosions, ulcers, and vesiculobullae • Anal: mucositis, erosions, and ulcers
Cutaneous	• Pleomorphic skin lesions tend to be targetoid vesiculobullae^2^ • Also described: vesiculobullous, targetoid, atypical target, papules, macules, or morbilliform morphologies^1^

RIME: reactive infectious mucocutaneous eruption.

**Table 3.** RIME and MIS-C diagnostic criteria.

RIME^2^	MIS-C^16^
• Evidence of an infectious trigger based on symptoms, imaging studies or lab tests; AND • At least two of: • Vesiculobullous/atypical target skin lesions affecting <10%; OR • Erosive mucositis involving two or more mucous membranes; OR • Non-contributory medication history	• An individual aged <21 years presenting with fever^a^, laboratory evidence of inflammation^b^, and evidence of clinically severe illness requiring hospitalization, with multisystem (⩾2) organ involvement (cardiac, renal, respiratory, hematologic, gastrointestinal, dermatologic, or neurological); AND • No alternative plausible diagnoses; AND • Positive for current or recent SARS-CoV-2 infection by reverse transcription–polymerase chain reaction, serology, or antigen test; or COVID-19 exposure within the 4 weeks prior to the onset of symptoms

RIME: reactive infectious mucocutaneous eruption; MIS-C, multisystem inflammatory syndrome in children; COVID-19, coronavirus disease 2019.

a Fever ⩾38.0°C for ⩾24 h, or report of subjective fever lasting ⩾24 h.

b Including, but not limited to, one or more of the following: an elevated C-reactive protein, erythrocyte sedimentation rate, fibrinogen, procalcitonin, D-dimer, ferritin, lactic acid dehydrogenase, or interleukin-6, elevated neutrophils, reduced lymphocytes, and low albumin.

There are multiple proposed immunomodulatory treatments for early/severe RIME (Table 4). In an adult randomized controlled trial (RCT) of SJS/TEN, ETN showed benefit compared to corticosteroids for healing time.^17^ Our patient received an initial dose of 0.6 mg/kg/dose SC based on the RCT dosing (Table 5) with a significant stabilization in mucocutaneous lesions that seemed to reverse when IVIG was administered within 24 h.^5,18,17^ Stabilization, when new skin lesions stop developing, is the goal of intervention, and thereafter, healing occurs naturally. We theorize that IVIG may have accelerated catabolism of ETN via saturation of the neonatal Fc receptor and neutralized ETN via anti-idiotypic antibodies that could bind to the Fc portion of ETN.^19–22^ Peak level of ETN occurs 2 days after injection.^22^ ETN is given after IVIG for resistant Kawasaki disease at doses of 0.8 mg/kg SC within 24 h then repeated at days 7 and 14 which was the dosing we adopted for our patient’s second dose.^23,24^ A second dose of ETN has been required when RIME continues to progress but in those cases (unlike our case), temporary stabilization prior to progression was not described.^5^

**Table 4.** Current reported immunomodulatory therapies for early and severe RIME.

Class	Drug
Immunomodulatory	• Systemic corticosteroids (early high-dose): ○ Prednisone^26^ ○ Prednisolone^13^ ○ Methylprednisolone^26,13^ ○ Dexamethasone^26^ • Steroid-sparing agents: ○ Cyclosporine^3,27^ ○ IVIG^9,10,28,29^ ○ Anti-TNF α agents: ■ Infliximab^4,30^ ■ Etanercept^5,17,18,30,31,32^

Source: Adapted from Ramien et al.^25^

IVIG: intravenous immunoglobulin; RIME: reactive infectious mucocutaneous eruption; TNF: tumour necrosis factor.

**Table 5.** Randomized control trials of etanercept dosing schedules used in treatment of RIME and related skin conditions.

Reference	Condition treated	Dosing
Eliades et al.^18^	SJS-TEN	0.8 mg/kg or 50 mg SC × 1–2 doses based on progression
Miller et al.^5^	RIME	0.6–0.8 mg/kg per dose for patient <60 kg or 50 mg for ⩾60 kg SC × 1 dose; second dose considered if worsening at 48 h
Wang et al.^17^	Drug-induced epidermal necrolysis	25 mg for patients <65 kg and 50 mg for those ⩾65 kg SC twice per week until improvement

RIME: reactive infectious mucocutaneous eruption; SJS-TEN: Steven–Johnson syndrome-toxic epidermal necrolysis; SC: subcutaneous.

MIS-C during the COVID-19 pandemic is a new concern in the age group commonly affected by RIME (Table 3). With inclusive criteria, many paediatric patients with an infectious prodrome may be presumed to have MIS-C and treated with IVIG, a key therapy component.^33^ However, in our case, there was an alternative plausible diagnosis so not all MIS-C criteria were met.

This report was limited by being unable to obtain a serum ETN level before repeated administration to prove our hypothesis that IVIG affected ETN levels. In addition, the natural history of RIME is to improve with good supportive care which our patient received. Despite our patient’s widespread skin involvement, early treatment initiation led to minimal and temporary dyspigmentation (common bothersome sequela for patients with richly pigmented skin).^1^

Dermatologists play an important role in diagnosing RIME promptly and working with multidisciplinary teams to confirm the diagnosis and deliver appropriate therapy. Our case highlights the overlapping features of RIME and MIS-C and the potential for treatment failure if both are simultaneously treated; that ETN efficacy can be recaptured with a second dose; and that early treatment can reduce chronic hyperpigmentation.
